# Elastance as a determinant of the effect of prone positioning on mortality in acute respiratory distress syndrome: a post hoc analysis of the PROSEVA trial

**DOI:** 10.1186/s13054-026-06060-3

**Published:** 2026-05-16

**Authors:** Ann A. Zalucky, Ewan C. Goligher, Jose Dianti, Andrew Willmore, Claude Guerin, Carolyn S. Calfee

**Affiliations:** 1 Department of Critical Care Medicine, Snyder Institute for Chronic Diseases, Cumming School of Medicine, Alberta Health Services, University of Calgary, Foothills Medical Center, Calgary, Canada; 2 Interdepartmental Division of Critical Care Medicine, University of Toronto, Toronto, Canada; 3 Department of Physiology, University of Toronto, Toronto, Canada; 4 Toronto General Hospital Research Institute, Toronto, Canada; 5 Department of Medicine, Division of Pulmonary, Critical Care, Allergy and Sleep Medicine, Cardiovascular Research Institute, University of California, San Francisco, CA United States of America; 6 Médecine Intensive-Réanimation, Hospital Edouard Herriot, Lyon, France; 7 University of Lyon, Lyon, France; 8 Institut Mondor de Recherche Médicale INSERM 955, ERL CNRS 700, Créteil, France

**Keywords:** Respiratory system elastance, Driving pressure, Ventilator induced lung injury, Prone positioning

## Abstract

**Background:**

Patient factors determining the benefit of prone positioning remain uncertain, resulting in the maneuver being applied indiscriminately among those with moderate-severe ARDS. We aimed to assess if baseline respiratory system elastance (Ers), or “stiffness”, determines the treatment effect of prone positioning on mortality.

**Methods:**

Bayesian logistic regression modeling of the PROSEVA Trial was used to estimate the posterior probability of prone positioning effect moderation by baseline Ers on 90-day mortality in patients with moderate-severe ARDS. As a secondary aim, we tested whether the absolute change in driving pressure of the respiratory system (∆DPrs ) in response to prone positioning predicted 90-day mortality, using logistic regression.

**Results:**

The treatment effect of prone positioning on mortality did not meaningfully vary with baseline Ers (posterior probability of benefit OR < 0.95 = 52%; interaction OR 0.94, 90% credible interval, CrI, 0.74–1.20). Higher baseline Ers was associated with greater improvements in DPrs at the end of the first prone session (β= -3.3, 95% confidence interval (CI) -4.09, -2.49; p = < 0.001). However, this response was not associated with mortality benefit in adjusted models (OR 1.14, 95% CI 0.96, 1.37; *p* = 0.14).

**Conclusions:**

The effect of prone positioning on mortality did not vary with Ers in the PROSEVA trial. Similarly, prone positioning-induced improvement in DPrs was not predictive of mortality in this cohort of passively ventilated ARDS patients.

## Background

Causal frameworks have proposed respiratory system elastance (Ers), the inverse of compliance, to be an important predictor of risk for ventilator induced lung injury (VILI) in patients with acute respiratory distress syndrome (ARDS) [1, 2]. Ers is inversely correlated with aerated lung volume and reflects the size of the ‘baby lung’ in ARDS [3]. Patients with reduced functional lung volume (small ‘baby lung’) have elevated Ers and are more susceptible to injurious pressures from mechanical ventilation [1].

For decades, prone positioning has been adopted as a rescue maneuver for severe hypoxemia, and evidence of its mortality benefit led to its use being broadly recommended among patients with moderate-severe ARDS (PaO_2_/FiO_2_ <150 mmHg) [4–6]. Patient factors determining the benefit of proning remain uncertain. Multiple studies have shown no association between improvements in oxygenation in response to proning and survival [7]. One mechanism by which prone positioning exerts physiological benefits is through the recruitment of open lung units [8]. By recruiting dependent dorsal lung regions and alleviating ventral hyperinflation, prone positioning reduces both static (trans-pulmonary plateau pressure) and dynamic (trans-pulmonary driving pressure (DP)) measures of lung stress [9]. Assuming patients with higher Ers have more atelectasis and more recruitable lung, they may respond preferentially to proning, independent of PaO_2_/FiO_2_, by improving the size of the ‘baby lung’ and attenuating VILI-mediated mortality risk.

We hypothesized that the treatment effect of prone positioning on mortality varies by baseline functional lung volumes as measured by Ers, whereby patients with increased Ers would respond preferentially to proning. To test this hypothesis, we conducted a secondary Bayesian analysis of the landmark PROSEVA randomized clinical trial. As a secondary aim, we tested whether the change inDP in response to proning was associated with mortality. We hypothesized that patients with a greater reduction in DP after proning would be associated with mortality benefit.

## Methods

This study was a post hoc analysis of the PROSEVA trial which has previously been published [6]. In brief, PROSEVA was a multicentre randomized trial which enrolled 466 patients with moderate-severe ARDS, randomizing 237 to undergo *≥* 16 h of prone positioning for up to 28 days and 229 to the supine position. The trial reported a significant decrease in all-cause 90-day mortality with prone positioning (17% absolute risk reduction, *p* < 0.001)^6^. We deployed a Bayesian multivariable logistic regression model to test the interaction between randomization to prone positioning and Ers on 90-day mortality, adjusting for age, PaO_2_/FiO_2,_ and severity as measured by the sequential organ failure assessment (SOFA) score. We applied an optimistic prior to prone positioning with moderate certainty (odds ratio (OR) 0.82, 95% credible interval (CrI) 0.45–1.47) [10], a neutral prior to the interaction term to express uncertainty about how treatment effect varies with elastance, and informative priors to all other covariates, as described previously [2, 11]. Sensitivity analysis using neutral priors for all covariates was conducted to assess their influence on model estimates. We hypothesized that patients with elevated Ers would have a larger mortality benefit to treatment (implying OR for interaction < 1). An interaction OR < 0.95 for the posterior distributions of the interaction term was set as the minimally clinically important threshold. As a secondary aim, we tested whether the absolute change in DP of the respiratory system (∆DPrs) or absolute change in Ers (∆Ers) in response to prone positioning [measured at: (i) 1-hr into the first prone session and (ii) at the end of the first prone session right before supination, compared to baseline] predicted 90-day mortality, using logistic regression adjusted for age, PaO_2_/FiO_2,_ SOFA score, and baseline DPrs or Ers respectively. We aimed to test whether DPrs and Ers would decrease in response to prone positioning and that this response would be associated with improved mortality.

Descriptive data are presented as median (IQR) for continuous variables, or count (%) for categorical variables. Between group differences were tested using student t-test, Wilcoxon-rank test, or χ2 test, respectively. Physiological responses to prone positioning compared to baseline were tested using paired t-test. Analyses were performed using R software [12]. All Bayesian models were computed using the brms package on a personal laptop computer and run using four chains and 2,000 iterations.

## Results

### Baseline physiological measurements

Data were available to compute Ers and DPrs in 443 patients (95%) at baseline. Among those randomized to proning, data was available to compute DPrs in 136 patients (57%) at 1-hr and 134 patients (56%) at the end of the first prone session. Baseline Ers and DPrs were similar between treatment and control groups (Table 1). Those without Ers or DPrs measurements were excluded from analysis. Proned patients included in the analysis were younger (55yrs (IQR 46,68) vs. 63yrs (IQR 47,75), *p* = 0.04), had a lower PaO_2_/FiO_2_ (97 mmHg (IQR 81,117) vs. 111 mmHg (IQR 97,134), *p* < 0.001), and a greater receipt of NMB (98% vs. 87%, *p* = 0.001) compared to those with incomplete data.

**Table 1:** Baseline characteristics and prone-induced changes in lung mechanics

	Prone position *N* = 221	Supine position *N* = 222	*p*-value
**Baseline**			
Age (yrs)	58 (46,72)	63 (51,73)	0.04
Male (%)	152 (69)	146 (66)	0.5
PaO_2_/FiO_2_ (mm Hg)	106 (86,124)	105 (86,123)	> 0.9
SOFA score	9 (8, 12)	10 (8,13)	0.01
NMB (%)	206 (94)	184 (83)	< 0.001
Ers (cm H_2_O/(ml/kg))	2.10 (1.80, 2.59)	2.11 (1.73, 2.64)	0.7
DPrs (cm H_2_O)	13 (11, 16)	13 (11, 16)	> 0.9
**1-hr into first prone session**			
Ers (cm H_2_O/(ml/kg))	1.84 (1.47, 2.29)	-	< 0.001*
DPrs (cm H_2_O)	11 (9, 14)	-	< 0.001*
ΔErs (cm H_2_O/(ml/kg))	-0.24 (-0.67, 0.03)	-	-
Percent ΔErs (%)	-12 (-34, 2)		-
ΔDPrs (cm H_2_O)	-2 (-5,0)	-	-
Percent ΔDPrs (%)	-14 (-32, 0)		-
**Pre-supination after the first prone session**			
Ers (cm H_2_O/(ml/kg))	1.73 (1.37, 2.15)	-	< 0.001*
DPrs (cm H_2_O)	11 (8, 13)	-	< 0.001*
ΔErs (cm H_2_O/(ml/kg))	-0.33 (-0.70, 0.00)	-	-
Percent ΔErs (%)	-15 (-30, 0)	-	-
ΔDPrs (cm H_2_O)	-2 (-4,0)	-	-
Percent ΔDPrs (%)	-20 (-30, 0)	-	-

Data are presented as median (IQR). *Paired t-test as compared to baseline physiological variable. SOFA= sequential organ failure assessment; NMB = neuromuscular blockade; Ers = respiratory system elastance; DPrs = respiratory system driving pressure; ΔErs = absolute change in respiratory system elastance; ΔDPrs = absolute change in respiratory system driving pressure. Absolute change was calculated by subtracting post prone physiological variable from baseline. Percent change was calculated by dividing the absolute change by the baseline value, multiplying by 100

### Respiratory system elastance

**Fig. 1:**
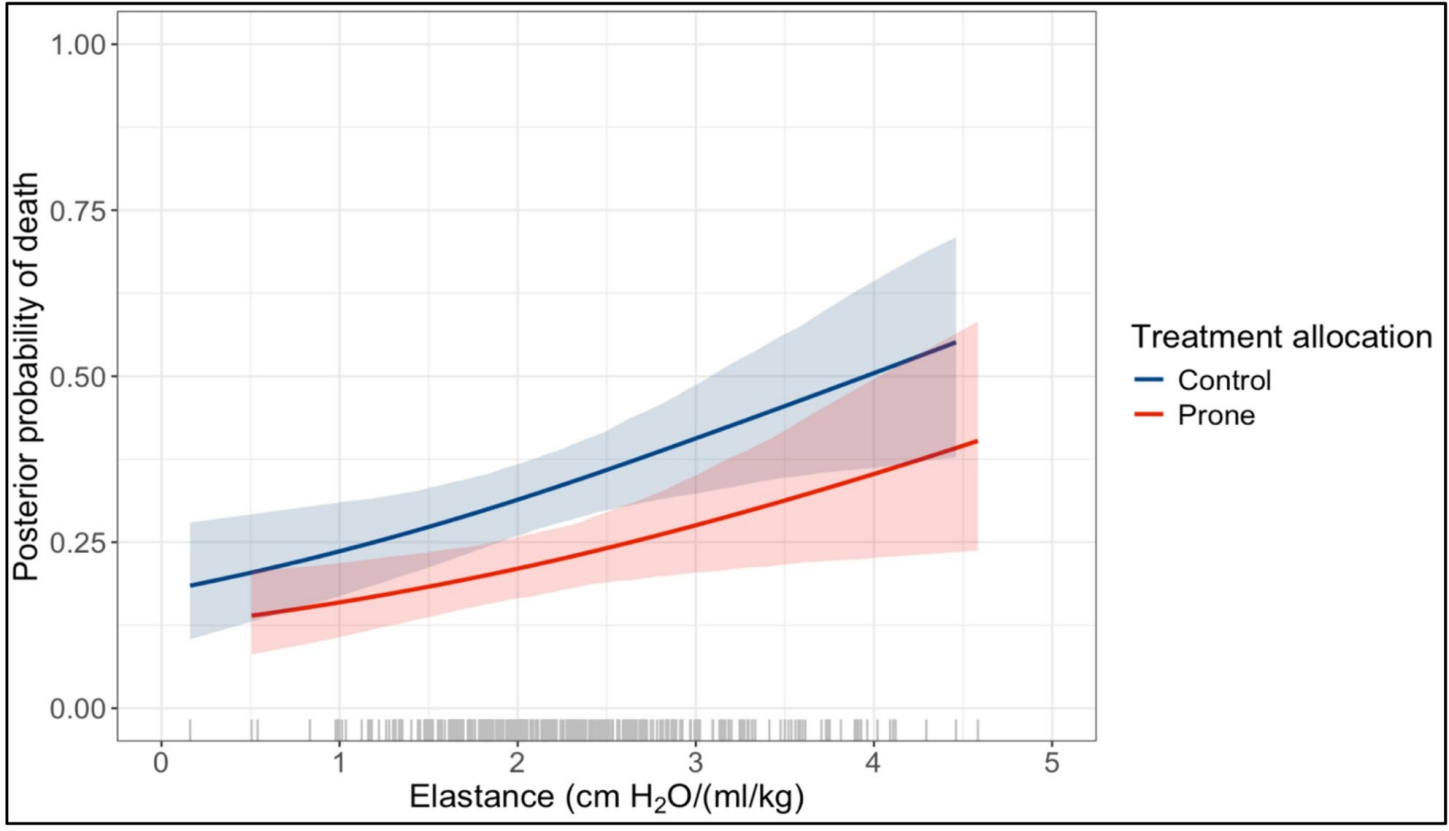
Treatment effect of prone positioning on all cause 90-day mortality according to baseline respiratory system elastance (Ers) (interaction OR 0.94, 90% CI 0.74–1.20); shaded areas represent 90% CI

The treatment effect of prone positioning on mortality did not meaningfully vary with baseline Ers (posterior probability of benefit OR < 0.95 = 52%; interaction OR 0.94, 90% credible interval, CrI, 0.74–1.20; Fig. 1). Sensitivity analysis using neutral priors for all covariates yielded similar results (interaction OR 1.31, 90% CrI 0.86–1.99).

### Physiological measurements in response to prone positioning

Among those randomized to the treatment arm, Ers and DPrs, on average, significantly decreased from baseline after prone positioning as measured at 1-hr and upon completion of the first prone session (Table 1). Higher baseline Ers was associated with greater improvements in DPrs after 1-hr (β=-3.4, 95% confidence interval (CI) -4.15, -2.62; *p* < 0.001) and at the end of the first prone session (β= -3.3, 95% CI -4.09, -2.49; p = < 0.001). However, neither the response in DPrsafter 1-hr (OR 0.94, 95% CI 0.79, 1.09; *p* = 0.4) nor following completion of the first prone session (OR 1.14, 95% CI 0.96, 1.37; *p* = 0.14) were associated with mortality in models adjusted for age, PaO_2_/FiO_2,_ SOFA score, and baseline DPrs. Similarly, there was no association between the response in Ers after 1-hr (OR 0.61, 95% CI 0.27,1.30; *p* = 0.21) and completion of the first prone sessions (OR 1.03, 95% CI 0.42, 2.64; *p* = 0.94) with mortality in adjusted models.

## Discussion

The effect of prone positioning on mortality did not vary with Ers, nor was the prone positioning-induced improvement in DPrs predictive of mortality in the current study. These findings suggest that although functional lung volume appears to increase with prone positioning, resulting in a net decrease in measures of dynamic tidal stress applied to the respiratory system, this mechanism does not appear to mediate the improved survival observed in the PROSEVA trial.

Regional distributions of transpulmonary forces across the lung - influenced by shape matching of the lung to chest wall and the effect of gravity on regional distribution of alveolar size - may be of greater importance in determining the effect of prone positioning on VILI risk than absolute changes in lung size [10]. The magnitude of the effect of prone positioning on regional lung stress distribution may be unrelated to the size of the lung available for ventilation (as reflected by Ers) and more determined by chest wall factors such as the distribution of abdominal weight and lung recruitability (which is not predicted by Ers) [13]. Unfortunately, the current study was limited by the absence of esophageal manometry and therefore further analysis on partitioned lung and chest wall mechanics could not be examined. Importantly, our results could be confounded by near universal use of NMB in the studied population. Patients receiving entirely passive lung-protective ventilation may be less susceptible to regional injurious lung-distending pressures through better shape matching preservation of lung to chest wall. Thus, these findings may not be generalizable to the spontaneously ventilated or non-intubated patient.

In summary, in this secondary analysis of the PROSEVA trial, baseline Ers did not determine the treatment effect of prone positioning on mortality in passively ventilated patients with ARDS. This interaction requires further study in the spontaneously breathing patient.

## Data Availability

Data for PROSEVA was made available by C.G. Data are however available from the authors upons reasonable request and with permission from C.G. ( [claude.guerin56@orange.fr](mailto: claude.guerin56@orange.fr) ).
